# Fragile X syndrome carrier screening in pregnant women in Chinese Han population

**DOI:** 10.1038/s41598-019-51726-4

**Published:** 2019-10-29

**Authors:** Chia-Cheng Hung, Chien-Nan Lee, Yu-Chu Wang, Chih-Ling Chen, Tze-Kang Lin, Yi-Ning Su, Ming-Wei Lin, Jessica Kang, Yi-Yun Tai, Wen-Wei Hsu, Shin-Yu Lin

**Affiliations:** 1 Sofiva Genomics Co., Ltd., Taipei, Taiwan; 2 Department of Obstetrics and Gynecology, National Taiwan University Hospital, Taipei, Taiwan; 3 Department of Obstetrics and Gynecology, National Taiwan University College of Medicine, Taipei, Taiwan; 4 Institute of Molecular Medicine, College of Medicine, National Taiwan University College of Medicine, Taipei, Taiwan; 5 Graduate Institute of Clinical Medicine, College of Medicine, National Taiwan University, Taipei, Taiwan; 6 Dianthus Maternal Fetal Medicine Clinic, Taipei, Taiwan

**Keywords:** Disease genetics, Molecular medicine

## Abstract

Fragile X syndrome (FXS) is the most frequent genetic cause of intellectual disability (ID). It was previously believed that the FXS prevalence was low in Chinese population, and the cost-efficiency of FXS carrier screening was questioned. This retrospective observational study was conducted between September 2014 and May 2017 to determine the prevalence of FXS carriers in a large Chinese cohort of pregnant women. The FMR1 CGG repeat status was determined in 20,188 pregnant Taiwanese women and we identified 26 women with premutation (PM). The PM allele was transmitted to the fetus in 17 pregnancies (56.6%), and six of 17 expanded to full mutation (FM). One asymptomatic woman had a FM allele with 280 CGG repeats. Prenatal genetic diagnosis of her first fetus revealed a male carrying a *FMR1* gene deletion of 5′ UTR and exon 1. Her second fetus was a female carrying a FM allele as well. This is so far the largest study of the FXS carrier screening in Chinese women. The prevalence of premutation allele for FXS in normal asymptomatic Taiwanese women was found to be as high as 0.13% (1 in 777) in this study. The empirical evidence suggests that reproductive FXS carrier screening in Taiwan might be cost-effective.

## Introduction

One of the major genetic causes of intellectual disability (ID), the fragile X syndrome (FXS) is an X-linked dominant disorder, where there is a defect in the fragile X mental retardation1 (FMR1) gene. Normally, in the 5′ untranslated region of the FMR1 gene, the cytosine-guanine-guanine (CGG) trinucleotide repeat is under 44^1^. In the case of full mutation (FM), with over 200 repeats, there would be abnormal methylation of the gene, and FMRP, which is crucial for brain development, cannot be produced^2^. If the repeat number falls between 55 and 200 (so-called premutation, PM), the carriers would have normal gene expression, but their children would have a higher risk of developing FXS^3^. Moreover, it is reported that PM carriers may present with fragile X-associated tremor/ataxia syndrome (FXTAS) and fragile X-associated primary ovarian insufficiency (FXPOI) in their late adulthood^4,5^.

Traditionally, Fragile X testing is performed by use of a laboratory-developed FMR1-specific PCR often followed by capillary electrophoresis. However, amplifying the entire CGG-rich template beyond about 100–130 repeats is challenging. In addition, differentiating full mutation from homozygous normal female samples has historically required a Southern blot reflex test, which is expensive, labor intensive and requires a large amount of high quality DNA^6^. With the advancement of PCR technology (FragilEase reagent kit, PerkinElmer Inc, Turku, Finland), however, the entire CGG repeat sequence in the FMR1 promoter region can be amplified to allow a confident detection of the trinucleotide repeats over 900^7^.

It was regarded that the FXS prevalence was low in Chinese population, and a routine screening of FXS carrier was unwarranted^6,8,9^. However, recent data has shown that the prevalence of PM and FM alleles was 1 in 883 in Hong Kong, similar to the prevalence found in Korea^7,10,11^. The objectives of this retrospective study were to determine the prevalence of FXS PM and FM carriers and the cost-effectiveness of the reproductive FXS carrier screening in a large Han Chinese female population.

## Materials and Methods

This was a retrospective observational study conducted between September 2014 and May 2017. This study was approved by the Institutional Review Boards of national Taiwan University Hospital (201706008RIND) since June 13, 2017. All samples were collected with informed written consent for the genetic test and all methods were carried out in accordance with relevant guidelines and regulations. Pregnant women at or above the age of 20 years were eligible for the study. Women with a known family history of FXS were excluded to avoid an overestimation of the premutation carrier rate in the general population. Women were tested on their own initiative and self-paid for the testing, which was not covered by the National Health Insurance of Taiwan. Pre-test counseling was given by obstetric clinicians at the first trimester, usually upon the first prenatal visit. A printed pamphlet containing information about fragile X carrier testing was provided. If the participants have more than 55 CGG repeats, genetic counseling by well-trained geneticists would be offered. The EDTA tube was used to draw two milliliters of peripheral blood from each participant.

The CGG repeat status of the FMR1 gene was determined using commercialized CGG repeat primed PCR (FragilEase reagent kit, PerkinElmer Inc, Turku, Finland). Testing was carried out based on manufacturer’s recommendations as previously mentioned^7^. In this assay, full-length and CGG repeat primed amplicons were produced using two gene-specific primers and were analyzed by capillary electrophoresis. Southern blot analysis was performed as described previously^6^.

Participants with positive test results (more than 55 CGG repeats) would be referred for genetic counseling. The post-test genetic counseling was provided to the pregnant women and her family members by a well-trained genetic counselor. The prognosis of a full-mutation or premutation carrier as well as the possible phenotypes of FXTAS and FXPOI was explained. After genetic counseling, prenatal and postnatal diagnosis could be made by via samples from amniocentesis, cord blood or neonatal blood. After the consultation, the patients arrived at their own decisions on whether to continue or terminate the pregnancies. We followed the ACMG Standards and Guidelines for fragile X testing. If the CGG repeats fall in the range from 45 to 54, it is regarded as intermediate and is not reported to the patients^12^.

The cost of the test from the society is estimated based on the expense under the National Health Insurance of Taiwan in this cohort. The method to calculate cost-effectiveness of the screening program is not a validated method.

## Results

**Figure 1:**
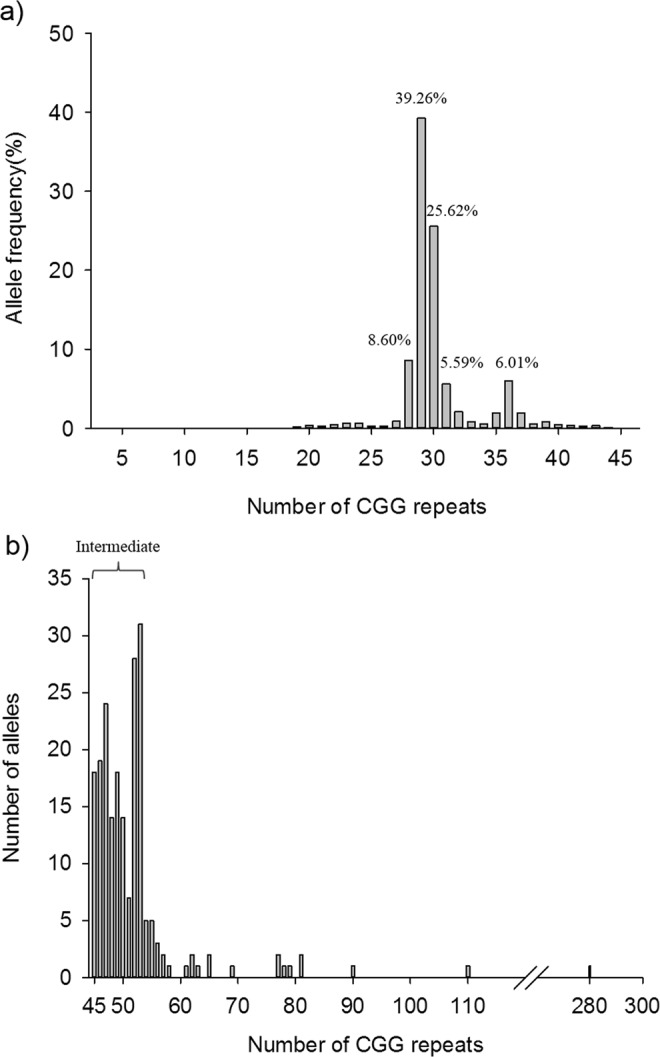
The CGG repeat distribution of the FMR1 gene in 20,188 individuals in Taiwan for (**a**) <45 CGG repeats and (**b**) 46–300 repeats.

Of the 20,188 pregnant Taiwanese women who underwent fragile X carrier testing, 19,982 of them (98.9%) were deemed normal (<45 repeats). The average age of the enrolled women was 31.7 years old, ranging from 20 to 54 years old. The CGG repeat ranged from 5 to 44 in normal FMR1 alleles, with the most prevalent alleles having 29 repeats (39.26%), followed by 30 repeats (25.62%), 28 repeats (8.60%) and 36 repeats (6.0%) (Fig. 1). There were a total of 178 (0.88%) women carrying intermediate alleles.

### Premutation

We also identified 26 out of 20,188 women with premutation (Supplementary Table S1). One pregnant woman had partial deletion of 5′ UTR and an upstream of (CGG)n repeats in FMR1 gene. Three of the PM carriers had two pregnancies each. The one had a twin pregnancy. There were 30 offspring information available (Table 1). Twenty-one of the twenty-six women carrying a PM allele received amniocentesis while the other five women chose to have genetic testing after delivery. The PM alleles were transmitted to the fetus in 17 pregnancies (56.6%), and six of them have expanded to FM. Table 2 summarized the consequences of the 17 transmissions of the maternal PM alleles. Most of the 17 PM alleles underwent unstable transmission with some changes in the repeat number. No maternal PM allele with 55 to 65 repeats expanded to FM. Six of the eight maternal alleles that exceeded 69 repeats expanded to FM.

**Table 1:** The offspring information from mothers carrying a PM or FM allele.

Mother	Sample Number	Fetus/Newborn					Total
		Gender	Normal	PM	FM	Deletion	
PM	26	Male	8	4	2		14
		Female	5	7	4		16
			13	11	6		30
FM	1	Male				1	1
		Female			1		1
Total	27		13	11	7	1	32

In the PM group, one woman had twin pregnancy, and three other women got pregnant twice during the study period. The lady with FM also got pregnant twice.

**Table 2:** Transmission of premutation or full mutation maternal allele to the offspring.

Maternal allele	Fetus/Newborn allele		
	CGG	Change in repeat	Untransmitted
CGG Repeats	Repeats	size	maternal allele
55	61	+6	40
56	56	+0	29
56	63	+7	31
57	57	+0	30
57	57	+0	30
61	61	+0	29
62	72	+10	29
62	81	+19	30
65	66	+1	29
69	245	+176	23
77	276	+199	32
78	83	+5	29
79	78	−1	29
81	200	+119	30
81	282	+201	23
90	280	+190	31
110	277	+167	31
280	283	+3	30

The eleven fetuses carrying PM alleles were delivered, while four of the six fetuses carrying FM alleles were terminated. The two fetuses with FM were both females and were delivered after the parents received comprehensive genetic counseling.

### Full mutation

One asymptomatic woman was found to have a FM allele with 280 CGG repeats. Tracing back to her family history, two of her male cousins had moderate intellectual disability and one female cousin had mild intellectual disability. None of them received genetic diagnosis. Her first pregnancy was terminated because prenatal genetic testing revealed a male fetus carrying a *FMR1* gene deletion of 5′ UTR and exon 1. Her second fetus was a female carrying a FM allele as well. After counseling, the parents decided to continue the pregnancy and deliver the female baby.

### Partial deletion

**Figure 2:**
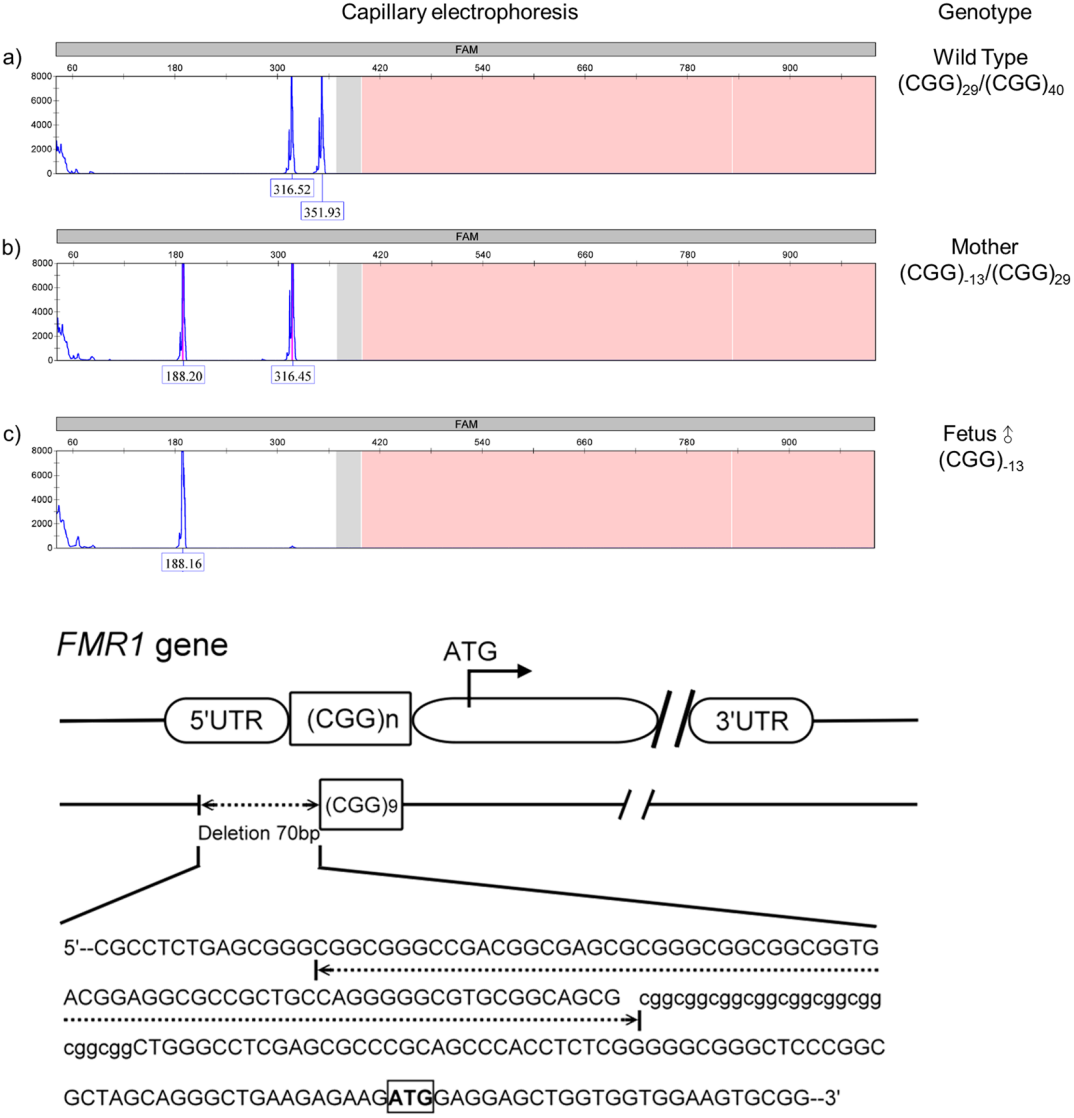
The capillary electrophoresis results of the FMR1 gene in (**a**) wild type, (**b**) mother with a partial deletion of one X chromosome, and (**c**) her male fetus who inherited the partial deletion. (**d**) Diagrammatic representation of a 70-bp deletion region in 5′UTR and the upstream CGG repeats in the FMR1 gene.

One asymptomatic women had a partial deletion of 5′ UTR and an unknown number of CGG repeats just upstream of FMR1 gene. By Sanger sequencing, we were able to confirm that this woman had one allele with 29 CGG repeats and the other allele with a 70 base-pair deletion of at 5′ UTR and nine CGG repeats (Fig. 2). The male fetus had inherited the deleted allele and was terminated.

### Cost analysis

In Taiwan, the fee of counseling is covered by National Health Insurance and each obstetrics appointment costs about USD $17 per visit. The cost for each genetic testing of FXS is about USD $ 125, regardless of blood or amniotic fluid samples. The test is self-paid by the patients. The fee for amniocentesis is about USD $100. In this study, a total of 20,214 tests for FXS was were performed, and 26 amniocenteses were done. In this cohort, it took about 118,885 US dollars to identify each woman carrying either a PM or FM allele. Taking the cost of amniocentesis and prenatal FXS genetic testing into consideration, the total cost to identify a fetus with FM is approximately 410,091 US dollars.

## Discussion

Population-based prevalence studies have been conducted in various countries. This is the largest study of the FXS carrier screening in Chinese women to date. The prevalence of premutation for FXS in normal asymptomatic Taiwanese women was as high as 0.13% (1 in 777) in this study. Our discovery corresponds with the recent study of Cheng *et al*., in which two women with PM and one with FM were identified in 2650 of Hong Kong’s Chinese pregnant women^7^. Their reported prevalence of PM and FM alleles was 0.11% (1 in 883). The reported PM carrier frequency in Korea and mainland China were 1 in 781 (0.12%), and 1 in 1113 (0.09%), respectively^10,11,13^. Our research results also show that FXS carriers are not at all rare in Chinese^6,8,9^. This study reported the highest female carrier frequency among Asian populations but still much lower compared to data from western countries such as Israel (1 in 113, 0.88%), Finland (1 in 246, 0.41%), Canada (1 in 259, 0.40%) and Australia (1 in 374, 0.27%)^14–18^. According to the ACMG position statement on prenatal/preconception expanded carrier screening, the mutation frequencies should be known in the population being tested for each screened disorder^12^. Therefore, the determination of FXS carrier rate in Taiwan is important before considering universal carrier screening.

Fragile X premutation carrier screening is now recommended for women with a family history of fragile X-related disorders or intellectual disabilities suggestive of fragile X syndrome, or women with a personal history of ovarian insufficiency^19^. It is a disease with a well-documented phenotype, an early-onset, and has detrimental effects on the quality of life, causing cognitive and physical impairment. Most women can accept screening for FXS carriers, as it is a simple maternal blood test. Meanwhile, after reproductive FXS carrier screening, the prenatal genetic diagnosis is available for FXS using the fetal DNA isolated from the amniotic fluid. Furthermore, once a carrier is identified, a family screening can be conducted to identify other carriers. Therefore, this screening has promising prospects, especially in modern society with fewer children born. Accordingly, the Royal Australian and New Zealand College of Obstetricians and Gynecologists recommends that the information on carrier screening for fragile X syndrome should be offered to all women planning pregnancy or in early pregnancy^20^.

There were two male fetuses born with FMR1 gene deletions in this cohort. The mother of the first fetus was clinically normal but had a family history of intellectual disability. She was identified to have a full FMR1 mutation with 283 CGG repeats. Hommond *et al*. reviewed 23 published cases with FMR1 gene deletions and reported an adolescent male with typical fragile X phenotype in whom a 300–400 bp deletion within exon 1 of FMR1 was found^21^. The patient’s clinically normal mother also has a full mutation with 700–900 CGG repeats. Our case may represent the second documented instance of a deletion in FMR1 arising from a full mutation so far. Our second case with FMR1 gene deletion inherited from his mother. The asymptomatic mother had a normal allele with 29 CGG repeats and another allele with a 70 bp deletion at 5′UTR end and nine CGG repeats (Fig. 2). Capillary electrophoresis initially revealed a negative value of CGG repeat numbers calculated by extrapolation. Southern blots failed twice to identify the deleted size of the mutated allele. With Sanger sequencing, however, we were able to confirm the 70 bp deletion at the 5′ UTR end and nine CGG repeats. Most reported cases with FMR1 deletions presented Fragile X syndrome or Fragile X syndrome-like phenotypes^21^. In fact, in addition to FMR1 promoter expansion, a small number of FXS cases (<1%) are caused by mutation in the coding region or deletion of the FMR1 gene^22^. Point, missense, nonsense, frameshift, and UTR region mutations have all been described^23–26^. These mutations account for approximately 4% of FXS patients meeting the clinical criteria for FXS but with a normal range of CGG repeats^23^. Therefore, detailed molecular analysis of the FMR1 gene may help to identify the exact mutation attributing to the FXS phenotype.

The CGG repeat distribution varies among different population. In our cohort, the most prevalent allele was 29 repeats (39.26%), followed by 30 repeats (25.62%), 28 repeats (8.60%) and 36 repeats (6.01%). Similar to the reports from Hong Kong and Korean, the most prevalent alleles were 29 and 30 repeats while the minor allele was 31 and 36 repeats, respectively^7,10,11^. In our study, five of the six maternal alleles that exceed 69 repeats expanded to FM, while in the Korean report, all of the maternal alleles over 70 repeats expanded to FM^3,11^. This information is important for genetic counselling of PM women picked up by the carrier screening programs, although an even larger sample size may be needed to confirm and to change clinical practice. Our finding is also compatible with previous literatures that an increased number of CGG repeats confer increased instability of alleles from one generation to the next, resulting in alleles with increased number of repeats in the progenies. (19% for 49–54 repeats, 30.9% for 55–59, and 80% for 60–65 repeats)^3^. It was reported that the proportion of female adults with at least one “low normal” CGG repeat allele (i.e., ≤26 repeats) was 35.0% in the adult carrier-screening cohort in Australia^18^. In our Chinese cohort, however, the proportion of the pregnant women with at least one allele with CGG repeats ≤26 was only 3.25%, and those carrying an allele with ≤20 repeats were only 0.83%. This discrepancy may highlight the importance of ethnicity in FMR1 allele distributions and frequencies.

There is no available data regarding the expenses of FXS treatment in Taiwan. In the United States and United Kingdom, the estimated life-cycle cost for an FXS patient is 615,397 US dollars and 510,900 US dollars, respectively^27,28^. It costs about 125 US dollars to do a FXS test in Taiwan. In this cohort, it took about 118,885 US dollars to identify a PM or a FM woman. Taking the costs of amniocentesis and prenatal FXS genetic test into consideration, the total cost of identifying a fetus with FM is approximately 410,091 US dollars. The empirical evidence suggests that reproductive FXS carrier screening might be cost-effective in Taiwan.

Last but not the least, pre-testing and post-testing prenatal genetic counseling can be challenging. The first difficulty in counseling is the variable phenotypes associated with FXS, especially in female fetuses with PM or FM^29^. X chromosome inactivation (XCI) is the phenomenon of the silencing of one of the two X chromosomes in mammalian females. Normally, XCI occurs randomly. However, preferential XCI can be observed under particular conditions. In the case of FMR1 mutation carriers, if the mutated chromosome is preferentially inactivated, FMRP can still be produced by the remaining normal allele, and the resulting phenotype would be less severe^30^. If a female fetus with FM is identified through prenatal diagnosis, Southern blot analysis should be applied to predict whether the fetus would be affected by FXS. In this cohort, only two female fetuses with FM were terminated, while the other two female fetuses with FM and seven female fetuses with PM were born. The second concern is that identification of a PM carrier may lead to anxiety regarding to the development of FXPOI and FXTAS^7^. The symptoms of FXTAS usually manifest in carriers over fifty years old, especially males^31^. Among female PM carriers, about 20% may experience early menopause before they turn 40, therefore targeted reproductive interventions are required^32^. Obstetricians, midwives, genetic counselors, and high-risk pregnant women should also be provided with medical information regarding this condition. Further referrals to psychiatric and neurologic specialists should be arranged as necessary.

There were some limitations in this study. Firstly, this study did not analyze the presence of AGG interruptions. The lack of AGG interruptions might aid in risk prediction of FM expansions from premutations with repeats <100^33^. Although testing for the AGG triplets is available clinically, it is not routinely performed in Taiwan and its clinical usefulness is yet to be determined according to the ACMG guideline^34^. Secondly, the enrolled pregnant women in this cohort were from local clinics and regional hospitals. As this a self-paid test not covered by the National Health Insurance, we could not exclude bias with respect to socioeconomic status.

## Conclusion

This is by far the largest study of the reproductive FXS carrier screening in Chinese women. The carrier rate of premutation for FXS in normal asymptomatic Taiwanese women was found to be 0.13% (1 in 777) in this study. One of the most impressive findings is that no PM mothers with CGG repeats in the low PM range (i.e., <65) have expanded to a full mutation. This could be important for genetic counselling of PM women picked up in the carrier screening programs. Larger sample size may be needed to confirm this before it could change clinical practice. The reported FXS carrier rate in Taiwan is important for prenatal counseling and for the implementation of universal screening as a public health policy.

## Supplementary information

Supplementary table S1

## Data Availability

All the data is available without restriction.
