# Supplementary material for: Fragile X syndrome carrier screening in pregnant women in Chinese Han population
Source: Sci Rep. 2019 Oct 29;9:15456. doi: 10.1038/s41598-019-51726-4 (PMC6820721; doi:10.1038/s41598-019-51726-4)
Supplement: Supplementary file 1 — Supplementary table S1 [file 41598_2019_51726_MOESM1_ESM.doc]

Fragile X syndrome screening in pregnant women in Chinese Han population

| Chia-Cheng Hung, Chien-Nan Lee, Yu-Chu Wang, Chih-Ling Chen, Tze-Kang Lin, Yi-Ning Su, Ming-Wei Lin, Jessica Kang, Yi-Yun Tai, Wen-Wei Hsu, Shin-Yu Lin |
| --- |

Supplementary table S1. Cases with PM or FM.

| **No.** | **Age** | **Birth date** | **Collection Date** | **Received Date** | **Allele 1** | **Allele 2** | **Follow up AF/Fetuses** | **Amniocentesis** | **Pregnancy outcome** |
| --- | --- | --- | --- | --- | --- | --- | --- | --- | --- |
| 1 | 32 | 1981/9/28 | 2014/9/26 | 2014/9/27 | 32 | 77 | 276 | yes | terminated |
| 2 | 30 | 1984/10/26 | 2014/11/30 | 2014/12/2 | 31 | 110 | 29/277 | yes | alive |
| 3 | 30 | 1984/12/12 | 2014/12/12 | 2014/12/12 | 29 | 78 | 1)29/83 2)29 | yes | alive |
| 4 | 29 | 1985-04-27 | 2015-03-29 | 2015-03-30 | 30 | 58 | 29 | yes | alive |
| 5 | 32 | 1982-09-28 | 2015-08-06 | 2015-08-07 | 29 | 63 | 30 | yes | alive |
| 6 | 32 | 1983-07-06 | 2015-09-12 | 2015-09-14 | 29 | 79 | 1)78 2)29 | yes | 1) alive 2) alive |
| 7 | 30 | 1984-12-23 | 2015-09-22 | 2015-09-24 | 40 | 55 | 37/61 | yes | alive |
| 8 | 28 | 1987-09-18 | 2015-11-16 | 2015-11-18 | 29 | 55 | 29/29 | Not done | alive |
| 9 | 38 | 1977-05-11 | 2015-12-10 | 2015-12-14 | 30 | 55 | 29/30 | yes | alive |
| 10 | 35 | 1980/11/2 | 2016/1/8 | 2016/1/19 | 23 | 69 | 1)30/245 2)23/30 | yes | 1) terminated 2) alive |
| 11 | 38 | 1978/2/11 | 2016/3/14 | 2016/3/15 | 29 | 62 | 72 | yes | alive |
| 12 | 32 | 1984/1/21 | 2016/4/2 | 2016/4/2 | 31 | 56 | 63 | yes | alive |
| 13 | 34 | 1982/3/31 | 2016/5/11 | 2016/5/12 | 30 | 57 | 1-1)29/57 1-2)28/29 | Not done | 1. alive 2) alive |
| 14 | 31 | 1984/8/16 | 2016/8/3 | 2016/8/3 | 29 | 56 | 29/56 | Not done | alive |
| 15 | 28 | 1988/8/31 | 2016/9/6 | 2016/9/7 | 29 | 65 | 66 | yes | alive |
| 16 | 31 | 1985/6/9 | 2016/10/3 | 2016/10/5 | 30 | 62 | 29/81 | yes | alive |
| 17 | 30 | 1986/8/22 | 2016/10/8 | 2016/10/11 | 23 | 81 | 31/282 | yes | alive |
| 18 | 27 | 1989/5/21 | 2016/10/10 | 2016/10/11 | 35 | 65 | 36 | yes | alive |
| 19 | 35 | 1981/2/22 | 2016/11/23 | 2016/11/24 | 31 | 90 | 29/280 | yes | terminated |
| 20 | 28 | 1988/2/5 | 2016/12/27 | 2016/12/29 | 29 | 61 | 30/61 | yes | O |
| 21 | 34 | 1982-01-16 | 2017-01-02 | 2017-01-04 | 30 | 81 | 200 | yes | terminated |
| 22 | 36 | 1980-10-01 | 2017-02-15 | 2017-02-16 | 29 | 56 | 29 | yes | O |
| 23 | 40 | 1976-09-25 | 2017-03-24 | 2017-03-24 | 28 | 55 | 29 | Not done | O |
| 24 | 26 | 1991-01-29 | 2017-04-18 | 2017-04-20 | 36 | 77 | 30/36 | yes | O |
| 25 | 27 | 1989-09-09 | 2017-05-08 | 2017-05-09 | 21 | 55 | 21 | O | O |
| 26 | 31 | 1986-06-10 | 2017-06-17 | 2017-06-17 | 30 | 57 | 29/57 | X | O |
| 27 | 27 | 1988-03-15 | 2015-06-08 | 2015-06-08 | 30 | 280 | 1)Deletion of 5'and exon 1 of FMR1 gene.  2)28/283 | yes | 1) terminated 2) alive |
